# Hispanic Secondary Stroke Prevention Initiative Design: Study Protocol and Rationale for a Randomized Controlled Trial

**DOI:** 10.2196/11083

**Published:** 2018-10-19

**Authors:** Olveen Carrasquillo, BreAnne Young, Stuti Dang, Orieta Fontan, Natalie Ferras, Jose G Romano, Chuanhui Dong, Sonjia Kenya

**Affiliations:** 1 Division of General Medicine Miller School of Medicine University of Miami Miami, FL United States; 2 Division of Geriatrics Miller School of Medicine University of Miami Miami, FL United States; 3 Department of Neurology Miller School of Medicine University of Miami Miami, FL United States

**Keywords:** Hispanics, Latinos, stroke, community health care, community health workers, randomized controlled trial, health care disparities, mobile phones, mHealth

## Abstract

**Background:**

Hispanic-Latino populations face a disproportionate stroke burden and are less likely to have sufficient control over stroke risk factors in comparison with other ethnic populations. A promising approach to improving chronic health outcomes has been the use of community health workers (CHWs).

**Objective:**

The objective of this randomized controlled trial is to evaluate the effectiveness of a CHW intervention among Latino patients at risk of recurrent stroke.

**Methods:**

The Hispanic Secondary Stroke Prevention Initiative (HiSSPI) is a randomized clinical trial of 300 Latino participants from South Florida who have experienced a stroke within the last 5 years. Participants randomized into the CHW intervention arm receive health education and assistance with health care navigation and social services through home visits and phone calls. The intervention also includes a mHealth component in which participants also receive daily text messages (short message service). The primary outcome is change in systolic blood pressure at 12 months. Other secondary outcomes include changes in low-density lipoprotein, glycated hemoglobin, and medication adherence.

**Results:**

Study enrollment began in 2015 and will be completed by the end of 2018. The first results are expected to be submitted for publication in 2020.

**Conclusions:**

HiSSPI is one of the first randomized controlled trials to examine CHW-facilitated stroke prevention and will provide rigorous evidence on the impact of CHWs on secondary stroke risk factors among Latino individuals who have had a stroke.

**Trial Registration:**

ClinicalTrials.gov NCT02251834; https://clinicaltrials.gov/ct2/show/NCT02251834 (Archived by WebCite at http://www.webcitation.org/72DgMqftq)

**International Registered Report Identifier (IRRID):**

RR1-10.2196/11083

## Introduction

### Background

With 795,000 annual cases, stroke is a leading cause of death and the single greatest cause of preventable adult disability in the United States [1]. Medical costs, medication, and lost productivity resulting from stroke cost an estimated US $34 billion annually [1]. Moreover, as our population ages, the stroke incidence is expected to increase dramatically. By 2035, an additional 3.7 million people are expected to have suffered from a stroke, resulting in a two-fold increase in health care costs [2].

Nearly 23% of stroke cases are recurrent strokes [1], and the risk of recurrence ranges from 14% to 40% within a 5-year period following the first attack [3]. Further, the adverse impact of recurrent stroke is more devastating than the initial attack; the fatality rate during the first 30 days after a recurrent stroke is more than double the fatality rate among first-time stroke victims [4]. Research indicates that the majority of recurrent stroke cases can be prevented by better control of modifiable risk factors [5]. However, Hispanic individuals (a term we used interchangeably with Latinos) face a disparate burden of stroke and stroke risk factors compared with non-Hispanic white individuals [6,7,8]; for example, hypertension, diabetes, and limited physical activity account for more strokes among Hispanic individuals than any other racial or ethnic group [9,10,11].

Hispanic individuals are also less likely to receive evaluation or treatment for hypertension or diabetes and are also less likely to maintain their treatment regimens after diagnosis [10,12]. Socioeconomic barriers such as lack of insurance, limited language, competing demands, and inadequate care access also disproportionately impact the Hispanic population, which further compound their health risks [13,14].

### Prior Work

A promising approach to improving disease outcomes among Hispanic individuals has been the use of community health workers (CHWs) [15]. CHWs are trusted members of a target community who understand the local health beliefs and recognize the social and historical experiences shaping their communities [15,16]. They serve as links between their communities and the health care system, acting as patient advocates, delivering prevention education and counseling services, and linking people to the appropriate care facilities [17]. There has been increasing evidence on CHWs for a variety of preventive and chronic conditions [16], including knowledge and behaviors aimed at vascular risk factors and self- management [18-20]. Although more limited, randomized studies have also shown that CHW interventions can improve physiological outcomes such as glycemic control [21].

Another approach of increasing interest in addressing disparities has been the use of mHealth devices and technologies. These approaches allow clinicians and researchers to provide health services through platforms such as short message service (SMS) text messaging, Web-based services, and dedicated phone apps [22]. With over 80% of minority adults having a cellular phone for phone calls or SMS text messaging [23], mHealth technologies are a potentially cost-effective way of delivering some health services to underserved populations [24,25].

Combined CHW interventions and mHealth approaches may be a potentially innovative approach to address the care of Hispanic stroke patients. Although there are increasing reports of mHealth-focused CHW project evaluations, the majority of studies are not randomized and provide limited information on the impact of the intervention [26]. One recent large randomized study among low-income patients with uncontrolled hypertension in Argentina reported that a CHW intervention that included SMS text messaging improved the proportion of patients, with controlled blood pressure increasing from 17.0% at baseline to 72.9% at 18 months in the intervention group and from 17.6% to 52.2% in the usual care group [27].

### Study Goals

To examine the impact of such an approach in the prevention of secondary stroke among Latinos, we designed a clinical trial consisting of a combined CHW and mHealth intervention. We examine the effectiveness of such a combined intervention in lowering of systolic blood pressure, which is the most important modifiable risk factor for recurrent stroke.

## Methods

### Overview and Conceptual Design

The Hispanic Secondary Stroke Prevention Initiative (HiSSPI) is a randomized single-blind, parallel-controlled trial of 300 Latino patients who have experienced an ischemic or hemorrhagic stroke in the last 5 years. The theoretical foundation of the HiSSPI study is grounded in the Chronic Care Model, an organizational framework for restructuring chronic disease management to create a partnership between health systems and communities [28]. The focus of our design emphasizes a community-based approach to successfully linking individuals in underserved populations with effective health care services [15,16]. The study was reviewed and approved by the University of Miami Institutional Review Board and is registered with ClinicalTrials.gov (NCT02251834).

### Study Setting

The study took place in Miami-Dade County, where Latinos compromise 68% of the population [29,30]. Unlike other parts of the country, the Latino population of the county is highly diverse, having ethnic origins from most countries in Latin America. As an example, Miami-Dade has the highest number of Cubans, Colombians, Hondurans, and Peruvians in the United States [31].

For this study, patients were recruited from the following 2 health systems in Miami’s Civic Center Health District: the University of Miami Health System (UHealth) and Jackson Health System. UHealth is a private, University-owned not-for-profit health care system, and Jackson Health System is a public safety-net hospital system. Both are quaternary care institutions and major teaching sites for the University of Miami Miller School of Medicine. Additionally, both systems are Florida-designated Primary Stroke Centers and see over 1000 stroke admissions per year combined.

### Study Participants: Inclusion and Exclusion Criteria

Eligible patients are Miami-Dade residents aged 18 or older, who self-identify as Hispanic or Latino, and who were admitted to the stroke service for an intracerebral hemorrhage or ischemic hemorrhagic stroke. Initially, the study was designed to recruit patients having had a stroke admission within the last month, but a year after the study began, inclusion criteria were expanded to any patient having had a documented admission for stroke within the last 5 years.

Because the study is aimed at preventing a secondary stroke, the focus was on stroke patients whose initial stroke was not very severe. Stroke severity is assessed using the modified Rankin Scale [32,33]. This scale evaluates a stroke patient’s degree of disability that resulted from the stroke. Patients can score on a range from 0 (no residual symptoms) to 6 (death). For study eligibility, patients must have an modified Rankin Scale score no greater than 3, which means they may have up to a moderate disability from the stroke but can still walk without assistance. Patients who have any immediate or life-threatening morbidity (eg, active cancer), those with an arm circumference greater than 47 cm (automated cuffs are unreliable at greater arm widths), or who are currently enrolled in another stroke, cardiovascular, or diabetes study were excluded from the study.

### Recruitment: Participant Selection

Recruitment began in February 2015 and will be carried out over the 5-year study period. Study recruitment uses a multimodal approach. One approach is having study coordinators track and review data on all stroke admissions at both Jackson Health System and UHealth. Study coordinators approach patients, describe the study in person while patients are in the hospital, provide interested participants with brochures detailing the study program, and collect detailed contact information. When not possible in person, this is done via phone after discharge.

As noted above, a year after the study began recruitment, inclusion criteria were expanded to any patient having had a documented admission for stroke within the last 5 years. This allowed coordinators to recruit from the stroke clinic through provider referrals. It also allowed for recruitment from an existing stroke registry. Patients enrolled in others stroke projects were also referred to us after they completed participation in their other studies.

### Consent and Enrollment

Identified participants are contacted via phone to assess their interest in participation, evaluate their study eligibility, and answer any questions. If the patient is interested and qualifies for participation, they are scheduled for a baseline appointment at the Clinical Research Center at the University of Miami. At the appointment, the study is again explained, and informed consent is obtained. Vital signs are measured and blood was collected, and a baseline questionnaire is subsequently administered. The project coordinator orally administers the survey and records participant responses into an electronic data management system (Research Electronic Data Capture) via laptop computer [34]. To ensure accuracy, baseline sessions are also audiorecorded and periodically reviewed for concordance with the data electronically entered.

The full baseline process takes approximately 90 minutes to complete. Participants receive US $50 compensation to cover the cost of travel and other incidentals once they have completed the full assessment. Upon completing the baseline, participants are randomized into 1 of 2 research arms by the project biostatistician.

### Randomization and Blinding

Patients are randomized at a 1:1 ratio to the enhanced care group or the CHW intervention group within each hospital. Within each site, every eligible patient receives a unique study subject identification number and the prespecified assignment that corresponds to the identification number through randomization. The principal investigator and the program clinical coordinator are blinded to study allocation. However, both participants and CHWs are aware of the group to which each participant was randomized.

### Control Arm: Enhanced Usual Care

Patients randomized into this group receive enhanced usual care. Depending on the stroke severity, patients at both facilities are either discharged home or to a short-term rehabilitation facility. Prior to release, the patient’s nurse or case manager ensures the patient is scheduled for a follow-up with their primary care provider and a neurologist. Patients lacking a primary care provider are provided with a list of potential follow-up care facilities and information on how to schedule an appointment.

For this project, participants in the control group also receive health education materials every 4 months including the following: “Lo que necesita saber sobre los ataques cerebrales,” a National Institute of Neurological Disorders and Stroke brochure that explains the causes of stroke, the associated risk factors, and strategies for prevention [35]; “Cómo prepararse para una cita con el medico,” a booklet that provides guidance, strategies, and tips to Latino patients on ways to better communicate with their physician [36]; and a National Heart, Lung, and Blood Institute bilingual Latino recipe cookbook [37].

### Community Health Worker Intervention

Through home visits and phone contact, CHWs empower patients with skills to manage their health including medication adherence, physical activity, nutrition, and mental well-being.

### Hiring and Training of Community Health Workers

The lead CHW for HiSSPI (OF) is a bilingual Florida-certified CHW [38] with several years of CHW experience. She has worked with our team on a previous diabetes intervention study [20]. In selecting additional CHWs to work on HiSSPI, we prioritize bilingual candidates with knowledge of the local community and prior experience in service delivery (eg, social, medical, education, consumer) to Latino populations. Other important selection criteria include maturity, communication skills, and prior positive work evaluations. CHWs also need to meet the basic requirements to be a Florida-certified CHW. A personal vehicle and valid driver’s license are also required.

For HiSSPI training, CHWs need to complete “Promoting Healthy Choices and Community Changes,” a 3-hour Web-based training provided by the Department of Health and Human Services Promotores de Salud Health Initiative [39]. This 4-part learning program gives CHWs health training on reaching vulnerable, low-income, and underserved members of Latino and Hispanic populations. For the cardiovascular disease-specific training, we use CHW training materials from the Centers for Disease Control and Prevention and National Heart, Lung, and Blood Institute as well as our own CHW training material [40-42]. Additional training on stroke and stroke prevention is also provided by the stroke neurologist on our team (JGR).

CHWs also receive ongoing training including continuing education modules on topics such as motivational interviewing, clinic and insurance navigation, cardiovascular disease care, and social support resource navigation. Additionally, weekly meetings are held with a CHW supervisor to obtain performance feedback and discuss individual cases. CHWs are further required to complete all University of Miami and National Institutes of Health required training in human subject research and HIPPA as well as specific research training that we developed for CHWs [43]. Textbox 1 provides additional details on these training modules.

### Intervention Enhancement Phase (Months 1-4)

Conceptually, we divided the CHW intervention into the following 2 phases: the enhancement phase, which consists of individualized health education, and the maintenance phase. The enhancement phase occurs during the first 4 months and includes home visits and phone calls. Although the number of home visits and phone calls is based on individualized decisions, as a rough, estimate we plan 5 home visits in this 4-month period and 2-3 phone calls per month. Although the length of calls is determined by CHW, it is expected that most calls would average under 15 minutes. In this phase, CHW helps the participant identify the issues that may affect his or her overall health and well-being. These can include direct influences, such as comorbid health conditions and behavioral risk factors, or more indirect influences, such as socioeconomic status and social context, poor health literacy, barriers in communication, and limited experience navigating the health care system. Once these barriers are identified, participants can then develop structured goals and methods for overcoming these obstacles in a manner that aligns with their needs and preferences.

CHW guides participants through this process by developing individualized health and well-being plans. This includes orienting participants on the principles of self-management and engaging them in a problem solving process that sets priorities for immediate problem resolution. To ensure that participants achieve their personal health goals regarding stroke risk and related risk factors, each CHW is tasked with a number of roles including, but not limited to, health and behavior counseling and coaching, medical service navigation (eg, scheduling appointments, sending reminders, providing guidance through the health system bureaucracy, etc), and social support (eg, identifying local social resources programs such as immigration services, tenant advocacy, and domestic violence programs). A major component of health education consists of blood pressure home self-monitoring. For participants who do not have a home blood pressure meter, CHWs help by providing one at no cost to them. However, data from these monitors will not be used for the outcome analyses. Those will be based on the blood pressure readings obtained during a structured assessment at 12 months, as described below.

### Maintenance Phase (Months 4-12)

During the maintenance phase, participants are expected to independently maintain progress on their patient navigation activities and individual lifestyle intervention goals. CHWs contact participants weekly by phone to check on their progress including the status of participant action plans, updates on lifestyle modifications, and addressing new problems that may have developed. Participants also initiate contact with CHWs when in need of additional support. Phone calls are also used for patient navigation purposes including reminding participants of their next doctor’s appointment and facilitating patient contact with their provider offices when needed. Home visits can also occur during this period to ensure participants are meeting their outlined goals. During the final contact, participants are notified that the intervention is concluding and that they will be contacted in the coming weeks to complete their follow-up visit at the University of Miami Clinical Research Center.

### Mobile Technology Component

The mHealth component of HiSSPI intervention is led by a telehealth expert (SD) in conjunction with the external vendor, GenerationOne [44]. The vendor uses a proprietary mHealth Connect platform that is compatible with most modern cell phones (including basic cellphones and smartphones) and carrier cell phone plans. It allows for messaging through either a Web browser or using standard SMS text messages. During outreach and subsequently during informed consent, participants are made aware that if randomized to the intervention, they would have the option of also participating in the mHealth component of HiSSPI. After the initial CHW home visit, participants are asked if they wish to participate in this component of the intervention. If the participant does not have a phone and wishes to participate, CHWs assist in helping them obtain a low-cost phone such as those offered through the federal Lifeline Program (if income eligible). Participants can also designate their primary caregiver or close relative to serve as their proxy for receiving messages and providing mHealth information.

Using the mHealth Connect software (GenerationOne, Inc, Southfiled, MI, USA), we developed a set of project-specific user-friendly query routines and informational tips sent to enrolled participants on a daily basis using a 61-day looping routine. The query routines include decision branching logic that allows the system to interact with the participant based on the information they provide. Responses from participants are recorded in a clinical dashboard where CHW is able to track and follow responses and trends for their assigned patients. In addition, CHWs receive alerts if their participant answers a question out of the expected predefined normal range. Participants can choose their preferred time to receive these daily questions.

**Description of Hispanic Secondary Stroke Prevention Initiative Community Health Worker training components.**

**University of Miami Basic Research Training:**

Collaborative Institutional Initiative Training

- Human Subjects Research
- Health Information Privacy

Research Electronic Data Capture Database Software Training

**Community Health Worker Certification:**

500 clock hours of formal work or volunteer experience providing community health worker services in any of the following domains of practice within the last 5 years:

- Communication and Education: tasks related to community and education
- Resources: tasks related to linking community members with available health and social services
- Advocacy: tasks related to advocating for the community’s health and social service needs

30 clock hours of content-specific training as follows:

- Communication and Education: 4 hours
- Resources: 4 hours
- Foundations of Health: 4 hours
- Professional Responsibility: 4 hours
- Electives (may related to any of the performance domains): 10 hours

**Department of Health and Human Services Office of Minority Services:**

Promotores de Salud Health Initiative: Promoting healthy choices and community changes

- Unit A: Understanding health decisions
- Unit B: Helping people make health choices
- Unit C: Understanding changes in the community
- Unit D: Health people make changes in the community

**Additional training:**

Florida Community Health Worker Coalition-Partnership to Train Community Health Workers in Patient-Centered Research (a 7-hour course on research training for community health workers developed with support from the Patient-Centered Outcomes Research Institute)

- Patient-centered outcomes research: rationale, definitions, role of community health workers
- Clinical trials: types, randomization
- Data collection methods: qualitative and quantitative methods, avoiding bias
- Informed consent process
- Study protocol and reporting: working in a research team
- Disseminating study results: to study participants, how community health workers can contribute to research manuscripts
- Ethics: Institutional Research Boards, privacy and confidentiality, professional boundaries

National Institutes of Health Community Health Worker Health Disparities Initiative Health Education Materials & Resources

- Su corazón, su vida: Manual del promotor y promotora de salud
- Healthy Heart, Healthy Homes series
- Salud para su Corazón: Bringing Heart Health to Latinos - A Community Program Guide for Latinos
- Approaches to Enhance Learning: Using Adult Learning and Popular Education with the National Heart, Lung, and Blood Institute Heart Health Curricula (webinar)
- Improving Heart Health with Community Health Workers, Promotores, and Community Educators (webinar)

An important component of the queries is that participants are asked to enter their blood pressure. These data are processed and organized into 3 response categories, namely, high risk (requires immediate attention, systolic blood pressure >180 mm HG), moderate risk (requires attention, systolic blood pressure >140 mm HG), and low risk or normal (systolic blood pressure <120 mm HG). These thresholds were based on existing guidelines at the time of the study [45] and clinician input (OC and JGR). For high-risk participants, the system alerts the patient to contact their physician or CHW; further, it sends the patient’s name to CHW so that they are aware that the patient requires a call that same day. In consultation with project physicians (OC, JGR, SD), CHWs decide a plan of action for such participants. This may include an emergency room referral.

Participants at moderate risk are provided with automated feedback. The data also alert CHWs of participants that may require additional follow-up. This information is valuable in helping CHWs further pinpoint the areas of concern, thereby creating a more focused and effective intervention strategy; for example, this may include calling participants and asking about medication adherence or recent changes in diet. When needed, CHWs can also alert the patients’ health care provider about patent blood pressure readings that remain high over several days. They can also help obtain an urgent appointment for such patients to see their primary care provider. CHWs and study team staff do not engage in medication management. When needed, they facilitate contact and interaction with the participant’s primary care provider who then decides if medication changes are needed.

Training of participants in the mHealth intervention is conducted by CHWs. The training includes how to interface with the study’s text support system, how to submit daily blood pressure readings, and how to use the various medication, diet, and physical activity reminders built into the system. In general, training of participants takes less than 60 minutes but varies depending on the patients’ baseline level of mobile phone use. Participant caregivers are also invited to participate in the training so that they may also be able to assist in the device usage or, in some cases, become the primary user of the technology for the participant.

With respect to data privacy, users are assigned a unique name for identifying and tracking user identity, and the server does not transmit or store any identifying information. In addition, user sessions are terminated after 15 minutes of inactivity. Data within the database server are encrypted using Transparent Data Encryption, which protects data at rest and data that are being transmitted over an electronic communications network using 256-bit encryption.

### Data Management

Survey data are collected on laptop computers connected to Research Electronic Data Capture [35], a secure cloud-based Web app for data capturing in both online and offline settings, managed by the University of Miami. On a monthly basis, data are reviewed by the study statistician for missing or out-of-range values and potential inconsistencies. As needed, these discrepancies are reviewed with the study team and statistician.

### Sample Size and Statistical Power

We reviewed stroke and cardiovascular intervention programs to estimate the sample size required for this study [21,46]. Sample size consideration and power analyses were performed based on the primary outcome variable of systolic blood pressure. Using an SD of 21 mm HG with an alpha significance level of.05 and two-sided *t* test analyses, we estimated that 150 participants per study arm (300 participants total) could detect a minimum difference of 8 mm HG between groups with 91% power. This is smaller than the 10 mm Hg threshold in secondary stroke prevention guidelines [47]. For our secondary outcome of low-density lipoprotein), we will have 80% power to detect a minimum low-density lipoprotein difference of 13 mg/dL given SD of 39 mg/dL. For adherence to antiplatelet or antithrombotic therapy, we will have 83% power to detect a 15% difference in adherence among the intervention versus control group. All data will be analyzed using the latest SAS software (Cary, NC).

### Primary Outcome

The primary outcome is a change in systolic blood pressure from baseline to the 12-month evaluation as measured using the Omron HEM-705CP automated oscillometric device (Lake Forest, IL, USA). Following the American Heart Association guidelines, 3 readings are taken, and the average of the last 2 readings is used as the blood pressure measurement [48]. The study is powered to test the hypothesis that at 1 year, systolic blood pressure of participants enrolled in the intervention will be 8 mm HG lower than those in the control (see below).

### Secondary Outcomes

Secondary outcomes are low-density lipoprotein, glycated hemoglobin (for diabetic participants), and adherence to antiplatelet or antithrombotic medications (for ischemic or embolic stroke patients). To assess low-density lipoprotein and glycated hemaglobin, a certified phlebotomist obtains 10 cc of blood. Samples are spun and delivered to the University of Miami Diabetes Research Institute for lipid profiling. Glycated hemaglobin analyses are performed via latex agglutination, and low-density lipoprotein is estimated using the Friedewald equation [49]. For those with triglycerides greater than 400 mg/dl, a direct low-density lipoprotein measurement is performed. Self-reported medication adherence is assessed using the Morisky Medication Adherence Scale [50].

### Confounding Variables

Potential confounders of our primary and secondary outcomes include sociodemographics (age, sex, income, and educational attainment); health insurance status; depression (Center for Epidemiological Studies Depression Scale) [51]; acculturation (modified Marin-Marin scale) [52,53]; health literacy (Short Assessment of Health Literacy-Spanish and English) validated in both English and Spanish) [54]; body mass index; and functional status (modified Rankin score) [32].

### Mechanistic Variables

Mechanistically, we expect the CHW intervention to be successful in blood pressure management through 2 primary pathways. The first is through better medication management. Although the intervention team is not involved in managing medications, our behavioral intervention can result in improved medication management through either increased medication adherence or by ensuring that patients have an appropriate and timely follow-up with their existing primary care providers who manage medications. The second is through lifestyle changes, primarily diet and exercise.

To conduct potential exploratory analyses of how the intervention may have resulted in improved outcomes, we are collecting data on potential mediators including medication adherence, medication intensification, salt intake, physical activity, and provider visits. This will allow us to examine the degree to which any improvements in blood pressure were mediated by changes in these variables. We are also collecting detailed data on service intensity. We can then examine the correlations between blood pressure control and items such as number of home visits, telephone calls, and group visit participation. Similarly, items in the cell phone intervention can also be correlated with blood pressure control, such as the number of days in which blood pressure values were uploaded or frequency of SMS text messaging responses. A summary of variables being collected is shown in Table 1.

### Statistical Analysis

The distribution of baseline values and outcomes will be examined for each arm. The intervention effects on outcomes (systolic blood pressure, low-density lipoprotein, and glycated hemaglobin) will be evaluated using linear regression models for continuous variables as a function of intervention status as well as with categorical classification variables to index time intervals pre- and postintervention. We will use statistical tests of the estimates of the regression parameters from this model to compare the major outcomes before and after the intervention groups in the full sample as well as between intervention groups. Adherence to antiplatelet or antithrombotic therapy will also be evaluated using logistic regression models as a function of intervention status. Covariates that will be included in the models will include age, sex, body mass index, and educational attainment. Relationships between the outcome variables and other potential covariates such as income, health insurance status, health literacy, and functional status will be explored to determine their potential inclusion in final models.

**Table 1:** List of variables and measures included in Hispanic Secondary Stroke Prevention Initiative baseline survey.

Variables		Measures or methods
**Primary outcomes**		
	Blood pressure [48]	OMRON HEM-705CP, validated oscillometric device
**Secondary outcomes**		
	Low-density lipoprotein [49]	Roche Cobas c501 (Friedewald equation), also registry
	Glycated hemaglobin	Bio-Rad D-10 Latex agglutination, also from registry
	Adherence to antiplatelet or antithrombotic medication [50]	Medication Adherence Scale
**Exploratory outcomes**		
	Quality of life [55,56]	EuroQual (NINDS CDE^a^ core)
	Visits to providers	Agency for Healthcare Research and Quality Medical Expenditure Panel Survey
	Hospitalization or stroke admissions [57]	Medical Expedinture Panel Survey and Northern Manhattan Study Questions
**Mechanistic variables (mediators)**		
	Blood Pressure Behaviors Compliance [58]	Hill Bone Scale (salt intake & blood pressure medication adherence)
	Fruit and Vegetable Intake [59]	Behavioral Risk Factor Surveillance System (as a very rough marker of any diet change)
	Medication Intensification [20]	Miami Healthy Heart Initiative Medication Intensification Criteria
	Physical Activity [60]	International Physical Activity Questionnaire, Common Data Elements
**Covariates (moderators)**		
	Sociodemographics	NINDS CDE (core)
	Acculturation [53]	Marin-Marin (5 items)
	Health Literacy [54]	Short Assessment of Health Literacy-Spanish and English
	Depression [51]	Center for Epidemiologic Studies Depression (NINDS CDE core)
	Body mass index	Genentech Stadiometer, Platform Scale
	Functional status [32,33]	Modified Rankin Scale (NINDS CDE core)

^a^NINDS CDE: National Institute of Neurological Disorders and Stroke Common Data Elements.

## Results

Funding for the study began on 2014 and the first participant was enrolled on February 26, 2015. Enrollment is planned to be completed by 2018, and a manuscript describing the baseline characteristics of the population is expected to be completed by 2019 and submitted for publication in 2020.

## Discussion

### Limitations

**Figure 1:**
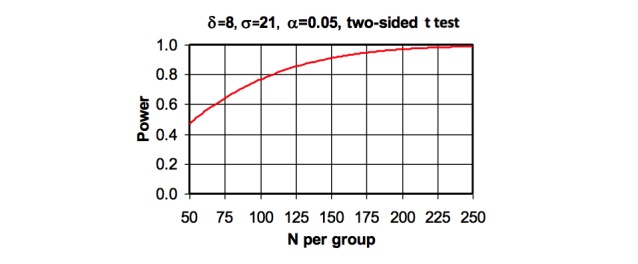
Statistical power and least detectable differences in Hispanic Secondary Stroke Prevention Initiative for systolic blood pressure.

Study attrition is a concern. In a prior diabetes study, we experienced 21% attrition [21]. As Figure 1 shows, even with such attrition, we would have >80% power to detect an systolic blood pressure difference of 8 mm HG. However, in that study, patients were younger and a much more geographically mobile group than stroke survivors. Thus, for HiSSPI, we expect an attrition rate of about 10%. Analytically, attrition will be handled by examining baseline characteristics of completers and noncompleters and testing patterns of attrition for randomness or ignorability. If the pattern of missing data is nonignorable, 1 approach is using baseline values carried forward. However, baseline values carried forward may not be the best method, and sensitivity analyses will be considered using different approaches such as weighted generalized estimating equations (using the inverse probability of dropout), multiple imputation, and propensity scores [61,62].

As noted above, Miami has a diverse Latino population of Caribbean, Central, and South Americans facing numerous distinct barriers to quality stroke care. Thus, it is an ideal location to generate findings that generalize to the US Latino population. In addition, initially, we also considered having Puerto Rico as another recruitment site. This site would allow us to test the intervention in a more homogenous Latino population where barriers such as immigration status and language are mitigated. Unfortunately, budgetary constraints did not allow us to include this site.

Lastly, a gap in the CHW literature is that few studies model the costs and benefits of CHW programs. Our proposed intervention of having one CHW assigned to a panel of 25-30 patients is costs approximately US $2000 per patient. Even though this figure pales in comparison with the cost of a subsequent stroke, of key importance to policy makers would be a formal cost-effective analysis. Although a formal cost-effective analysis is not part of the protocol, as part of the survey instrument, we will be collecting quality of life data using a standardized health state instrument (EQ5) and collecting data on health care utilization and hospitalizations. If linked to expenditure data, such data would allow for a future cost-effective evaluation.

### Conclusions

Results collected from HiSSPI will provide important information on the effectiveness of a combined CHW and mHealth intervention on recurrent stroke risk among Hispanic-Latino populations. The study highlights the innovative role CHWs play in chronic care delivery, and our findings will further gauge the feasibility of this framework in the existing health care system using mobile technologies, which may be more scalable options for chronic disease management.

## Abbreviations

CHW: community health worker
HiSSPI: Hispanic Secondary Stroke Prevention Initiative
NINDS CDE: National Institute of Neurological Disorders and Stroke Common Data Elements
SMS: short message service
UHealth: University of Miami Health System
